# Can vertical separation of species in trawls be utilized to reduce bycatch in shrimp fisheries?

**DOI:** 10.1371/journal.pone.0249172

**Published:** 2021-03-26

**Authors:** Roger B. Larsen, Bent Herrmann, Jure Brčić, Manu Sistiaga, Kristine Cerbule, Kåre Nolde Nielsen, Nadine Jacques, Mark J. M. Lomeli, Adnan Tokaç, Elsa Cuende

**Affiliations:** 1 UiT, The Arctic University of Norway, Tromsø, Norway; 2 SINTEF Ocean, Trondheim, Norway; 3 Department of Marine Studies, University of Split, Split, Croatia; 4 Institute of Marine Research, Bergen, Norway; 5 Norwegian University of Science and Technology, Trondheim, Norway; 6 Pacific States Marine Fisheries Commission, Newport, Rhode Island, United States of America; 7 Faculty of Fisheries, Fish Capture and Processing Department, Ege University, Izmir, Turkey; 8 AZTI, Marine Research, Basque Research and Technology Alliance (BRTA), Bizkaia, Spain; Tanzania Fisheries Research Institute, UNITED REPUBLIC OF TANZANIA

## Abstract

Several shrimp trawl fisheries use a Nordmöre sorting grid to avoid bycatch of fish. However, small fish can pass through the grid. Therefore, the retention of juvenile fish often remains an issue during shrimp trawling. We investigated the vertical distribution of deepwater shrimp (*Pandalus borealis*) and dominant bycatch species at the point where the Nordmöre grid section is installed. This was achieved using a separator frame which split the net vertically into three compartments of equal entry size. Our results showed that shrimp predominately follow the lower part of the trawl belly, whereas species such as redfish (*Sebastes spp*.), cod (*Gadus morhua*), polar cod (*Boreogadus saida*) and American plaice (*Hippoglossoides platessoides*) preferred the mid-section in the aft of the trawl. Haddock (*Melanogrammus aeglefinus*) primarily entered through the upper section of the trawl belly. Using these results, we predict that a vertical separation device installed forward of a 19 mm Nordmöre grid combined with a 35 mm codend would result in a significant reduction in bycatch with only minor loss of shrimp.

## Introduction

The deep-water shrimp (*Pandalus borealis*) has a widespread distribution in the North Atlantic and the North Pacific Oceans and is a commercially important species in many countries [1]. Since 1985, the global landings for this species have varied between 200 000 and 450 000 tons, which makes it one of the most important crustacean fisheries worldwide [2].

Deepwater shrimp and other species in the family Pandalidae live most of their life relatively close to the seabed [3,4] and are almost exclusively harvested with bottom trawls. The use of this gear results in excessive bycatch of juvenile fish and other marine organisms [5,6]. Mitigating shrimp trawl bycatch has been the subject of extensive research efforts over the last decades and represents significant management challenges [7–10]. The introduction of bycatch reduction devices has improved the selectivity of shrimp trawls and led to a reduction in the catch of undersized shrimp and unwanted species while avoiding excessive loss of legal-size shrimp.

**Fig 1:**
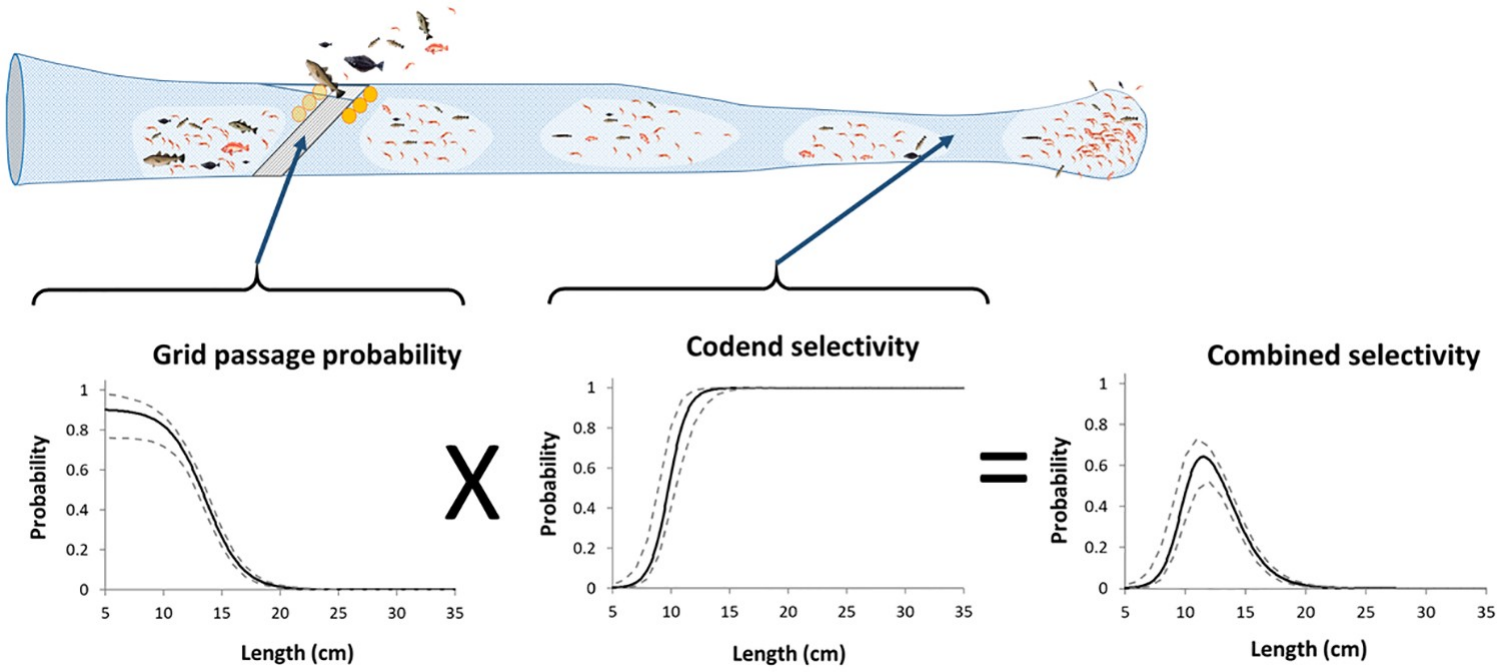
Combined selectivity process of a shrimp trawl. The figure explains the working principles and selection curves for the Nordmöre grid and the codend.

The implementation of the Nordmöre grid in the early 1990s has led to significant reductions of fish bycatch in shrimp fisheries in the North Atlantic [11–14]. In the Barents Sea deep-water shrimp fishery, the use of a Nordmöre grid with a maximum bar spacing of 19 mm and a selective codend with a minimum mesh size of 35 mm is compulsory (Fig 1). However, juvenile fish such as cod (*Gadus morhua*), redfish (*Sebastes spp*.), haddock (*Melanogrammus aeglefinus*), polar cod (*Boreogadus saida*) or American plaice (*Hippoglossoides platessoides*) are still often caught in large numbers [15–17]. Most of the very small fish entering the gear pass between the vertical bars of the grid but are subsequently released through the codend meshes. A fraction of the medium sized fish entering the gear are able to pass between the bars of the grid, however, they are too large to subsequently be released through the codend meshes, and therefore are retained by the gear. Fish that are larger in size than the spaces between the vertical bars of the Nordmöre grid are prevented from entering the codend, i.e. they escape at the grid. Thus, for these fish bycatch species the combined retention probability curves where a grid and codend configuration are used become bell-shaped and have a species-specific size interval of high retention risk [18] (Fig 1).

Several gear modifications have been tested to improve the selectivity of shrimp trawls. These include additional grids or sieve panels [15,19], various codend designs regarding mesh type [16,17], lights [20], and grid designs [21]. However, the results from these trials were variable and, therefore, more research is required to determine a design that reduces bycatch while minimising the loss of target animals.

Previous research has shown that species-specific behaviour in trawls can be used for species separation that allows some of them to escape the trawl [22,23]. For example, Norway lobster (*Nephrops norvegicus*) are located in the lower portion of the trawl, allowing fishers to modify their gear to separate the target species and sizes from unwanted bycatch and improve trawl efficiency [24–27]. Based on these results, the aim of the current study was to investigate improvements in species and size selectivity in the Barents Sea deepwater shrimp trawl fishery by applying a vertical separation in front of the compulsory Nordmöre grid section. Specifically, we aimed to quantify: 1) the vertical distribution of deepwater shrimp and finfish bycatch species; and 2) potential reduction in finfish bycatch that could be achieved through the separation of catch before it meets the Nordmöre grid.

## Materials and method

### Ethics statement

This study did not involve endangered or protected species. Experimental fishing was done on board a research vessel in accordance with the fishing permit granted by the Norwegian Directorate of Fisheries (18/14793). This fishing permit allows catches of shrimp and fish to be landed. No other permit was required to conduct this study.

### Vessel, area, time, and gear set-up

The fishing trials were performed on board the research vessel “Helmer Hanssen” (63.8 m LOA, 4080 HP) from the 13th to the 14th of December 2019. The fishing ground was located in the northern part of the Barents Sea, west of Spitsbergen (79°02.88N 10°24.32 E). The trials were carried out with a Campelen trawl, which was 1800 meshes (40 mm mesh size) in circumference. The main body of the trawl was constructed with 2 mm diameter (Ø) polyethylene netting with a mesh size of 80 mm in the wings and 40 mm in the belly. We used a set of Thyborön 7A-8 (7.4 m^2^, 1750 kg) trawl doors, and a 20 m long “strapping rope” of Ø20 mm (linked between the warps, 80 m ahead of the doors) to limit the door-spread to 48–52 m while towing. The trawl was connected to the trawl doors with 40 m bridles and sweeps. We used a rockhopper groundrope which was 19.2 m in length and composed of three sections, each equipped with 46 cm rubber discs that were attached to the fishing line (19.2 m in length). The wingspread and headline height were measured by Scanmar sensors to 15 m and 6.5 m, respectively.

**Fig 2:**
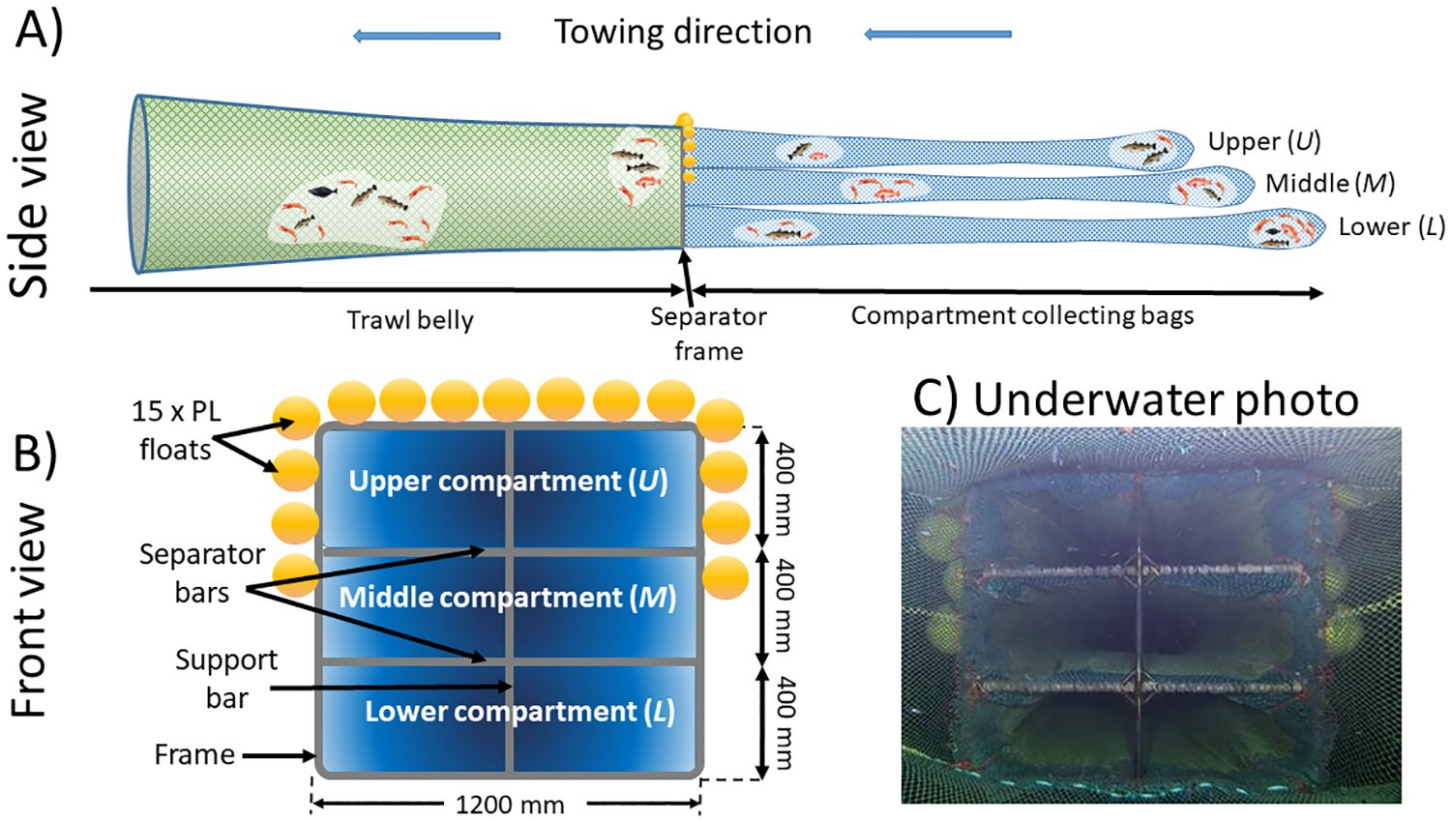
The experimental design. (A) the separator frame divided into three compartments and collecting bags, (B) the construction details of the frame, and (C) an underwater photo of the frame.

We replaced the Nordmöre grid section and the codend with a rectangular separator frame (Fig 2). The frame was horizontally divided into three equal-sized compartments (lower, middle and upper) each with a catch-collecting codend. Each compartment was 400 mm high and 1200 mm wide. The rectangular separator frame was built using steel rods (Ø20 mm) and 15 plastic floats (PL Ø200) to maintain the correct orientation during trawling. A 50 mm mesh size codend (collection bag) with a 6 mm mesh size inner net was attached to each compartment (Fig 2).

The haul duration was standardized to 60 min. After each haul, the catch from each compartment was sorted by species and the length of all fish bycatch below 40 cm in length was measured because fish larger than 40 cm in length are unlikely to pass through a Nordmöre grid with a 19 mm bar spacing. Fish length was measured to the nearest 0.5 cm below. Where a large number of a single species was caught (i.e., > 1000 individuals), the catch was subsampled. Where possible, a 1 kg of deepwater shrimp catch was subsampled, and their carapace length was measured to the nearest 0.5 mm below using callipers.

### Data analysis and parameter estimation

The probability of any species entering each compartment is a function of size. The number of individuals (n) of any species in each length class (*l*) caught in haul *i* in the lower (*L*), middle (*M*) and upper (*U*) compartments was represented as *nL*_*li*_, *nM*_*li*_, and *nU*_*li*_, respectively. The expected probability of capture for any species in the lower (*EPL*_*li*_), middle (*EPM*_*li*_) and upper (*EPU*_*li*_) compartment is calculated as:

$$\begin{array}{c} {EPU}_{l}=\frac{\sum_{i=1}^{h}\left\{\frac{{nU}_{li}}{{qU}_{i}}\right\}}{\sum_{i=1}^{h}\left\{\frac{{nL}_{li}}{{qL}_{i}}+\frac{{nM}_{li}}{{qM}_{i}}+\frac{{nU}_{li}}{{qU}_{i}}\right\}}\\ {EPM}_{l}=\frac{\sum_{i=1}^{h}\left\{\frac{{nM}_{li}}{{qM}_{i}}\right\}}{\sum_{i=1}^{h}\left\{\frac{{nL}_{li}}{{qL}_{i}}+\frac{{nM}_{li}}{{qM}_{i}}+\frac{{nU}_{li}}{{qU}_{i}}\right\}}\\ {EPL}_{l}=\frac{\sum_{i=1}^{h}\left\{\frac{{nL}_{li}}{{qL}_{i}}\right\}}{\sum_{i=1}^{h}\left\{\frac{{nL}_{li}}{{qL}_{i}}+\frac{{nM}_{li}}{{qM}_{i}}+\frac{{nU}_{li}}{{qU}_{i}}\right\}} \end{array}$$ (1)

Where *h* is the number of hauls conducted and *qL*_*i*_, *qM*_*i*_ and *qU*_*i*_ are the fraction of measured individuals (sampling factor) in haul, *i*, in each compartment. The averaged compartment entry probability curves, *EPL(l*, ***vL****)*, *EPM(l*, ***vM****)* and *EPU(l*, ***vU****)* were estimated by pooling the data across hauls and described using parametric models, where ***vL***, ***vM*** and ***vU*** are vectors of parameters from the respective models. Therefore, the analysis was reduced to a maximization problem to estimate the values of the parameters ***vU***, ***vM*** and ***vL***, which made the observed experimental data averaged over hauls most likely:

$$\begin{array}{c} -\sum_{l}\sum_{i=1}^{h}\left\{\frac{{nU}_{il}}{{qU}_{i}}\times ln(EPU(l,vU))+(\frac{{nL}_{il}}{{qL}_{i}}+\frac{{nM}_{il}}{{qM}_{i}})\times ln(1.0-EPU(l,vU))\right\}\\ -\sum_{l}\sum_{i=1}^{h}\left\{\frac{{nM}_{il}}{{qM}_{i}}\times ln(EPM(l,vM))+(\frac{{nL}_{il}}{{qL}_{i}}+\frac{{nU}_{il}}{{qU}_{i}})\times ln(1.0-EPM(l,vM))\right\}\\ -\sum_{l}\sum_{i=1}^{h}\left\{\frac{{nL}_{il}}{{qL}_{i}}\times ln(EPL(l,vL))+(\frac{{nM}_{il}}{{qM}_{i}}+\frac{{nU}_{il}}{{qU}_{i}})\times ln(1.0-EPL(l,vL))\right\} \end{array}$$ (2)

where the summations were over length classes *l* and *h* hauls. A sufficiently flexible model for *EPX*(*l*, ***vX***), where *X* represents *L*, *M* or *U*, respectively, was required to describe the main trends in the experimental data for the different species. Eq 1 is often applied in catch-comparison studies to estimate the efficiency/selectivity of fishing gears [28,29] and this model was adapted to model *EPX*(*l*, ***vX***) thus:

$$EPX(l,vX)=\frac{exp(f(l,vX))}{1.0+exp(f(l,vX))}$$ (3)

Where *f* is a polynomial of order *k* with coefficients *vX*_*0*_,*…*,*vX*_*k*_ so ***vX*** = (*vX*_*0*_,*…*,*vX*_*k*_). *EPX*(*l*, ***vX***) expresses the probability of finding a deepwater shrimp or fish of length class *l* in compartment *X* given that it was observed in one of the three compartments. A value of 0.33 for *EPX*(*l*, ***vX***) implies that the compartment entry probability corresponds to the fraction of the entry area that compartment *X* takes of the total entry area. In this case, where the total entry area is separated in three equal compartments (see Fig 2), each compartment comprises 33% of the total area, hence the value of 0.33. In that case, the species does not exhibit behavioural preference [26] for that specific compartment. In contrast, a high value would show preference for a specific compartment. We used the following formula for *f* (*l*, ***vX***).

$$f(l,vX)=\sum_{i=0}^{4}{vX}_{i}\times {\left(\frac{l}{100}\right)}^{i}={vX}_{0}+\;{vX}_{1}\times \;\frac{l}{100}+{vX}_{2}\times \frac{l^{2}}{100^{2}}+\cdots +v_{X4\;}\times \;\frac{l^{4}}{100^{4}}$$ (4)

We considered *k* of up to an order of 4 with parameters ***vX*** = (*vX*_*0*_,*…*,*vX*_*4*_) as our experiences from prior studies have demonstrated that this provides a model that is sufficiently flexible for modelling the vertical separation for different species [26,27,30]. By omitting one or more of the parameters *vX*_*0*_*…vX*_*4*_ in Eq 4, 31 potential models describing *EPX*(*l*, ***vX***) were obtained. Model averaging was applied to describe *EPX*(*l*, ***vX***) [31,32]. In the resulting combined model, the individual models were ranked and weighted according to their AIC values [31]. Models yielding AIC values within +10 of the value obtained by the model with the lowest AIC [33] were considered to contribute to *EPX*(*l*, ***vX***) based on the procedure described by Katsanevakis [34] and Herrmann et al. [32]. The ability of the combined model to describe the experimental data was assessed based on the *p*-value, which expresses the likelihood of obtaining a discrepancy at least as large as that observed between the fitted model and the experimental data by chance. Therefore, in order to accept a combined model, the *p*-value had to be > 0.05 [35]. In the case of poor fit statistics (*p*-value < 0.05; deviance >> DOF (Degree Of Freedom)), the deviations between the experimentally observed data and the fitted curve were examined to determine whether the difference was due to structural problems when the experimental data was described using the combined model, or data over-dispersion.

Confidence intervals (CI) for *EPX*(*l*, ***vX***) were estimated using a double bootstrap method within the software SELNET [36]. The procedure accounted for uncertainty due to between-haul variation in the compartment entry probability by selecting *h* hauls with replacement from the *h* hauls available during each bootstrap repetition. The within-haul uncertainty in *EPX*(*l*, ***vX***) was accounted for by randomly selecting deepwater shrimp or a given fish species with replacement from each size class. The number of fish or deepwater shrimp selected from each size class was the number of shrimp or a given fish species with that length being measured in that haul summed over compartments. In case of subsampling in the specific haul the resampling of the length class data was conducted prior to raising the data by the subsampling factor. This enables to account for increased uncertainty due to subsampling [37]. The data were then combined, as described above, and the length-dependent entry probability for each vertically separated compartment was estimated. In total, 1000 bootstrap replicates were performed and the Efron 95% CI [38] was calculated for each compartment entry probability curve. Incorporating the combined model approach described above in each of the bootstrap replicates allowed us to consider additional uncertainty regarding the compartment entry probability due to uncertainty in model selection [29].

In addition to quantifying the length-dependent probability to enter a specific compartment (lower, middle or upper), the probability to enter one of the two lower compartments (lower + middle) was quantified using the following formula:

EPL(l,vL)+EPM(l,vM)=1.0-EPU(l,vU) (5)

### Estimation of the length-integrated compartment entry probability

An average value for the compartment entry probability that does not consider the size of the species is obtained by summing over length classes. These are denoted length-integrated average values for each of the compartment entry probabilities and can be estimated by:

$$\begin{array}{c} {EPU}_{average}=\frac{\sum_{l}\sum_{i=1}^{h}\left\{\frac{{nU}_{li}}{{qU}_{i}}\right\}}{\sum_{l}\sum_{i=1}^{h}\left\{\frac{{nL}_{li}}{{qL}_{i}}+\frac{{nM}_{li}}{{qM}_{i}}+\frac{{nU}_{li}}{{qU}_{i}}\right\}}\\ {EPM}_{average}=\frac{\sum_{l}\sum_{i=1}^{h}\left\{\frac{{nM}_{li}}{{qM}_{i}}\right\}}{\sum_{l}\sum_{i=1}^{h}\left\{\frac{{nL}_{li}}{{qL}_{i}}+\frac{{nM}_{li}}{{qM}_{i}}+\frac{{nU}_{li}}{{qU}_{i}}\right\}}\\ {EPL}_{average}=\frac{\sum_{l}\sum_{i=1}^{h}\left\{\frac{{nL}_{li}}{{qL}_{i}}\right\}}{\sum_{l}\sum_{i=1}^{h}\left\{\frac{{nL}_{li}}{{qL}_{i}}+\frac{{nM}_{li}}{{qM}_{i}}+\frac{{nU}_{li}}{{qU}_{i}}\right\}} \end{array}$$ (6)

Where the outer summation covers the length classes caught during the trial.

By incorporating Eq (6) into each of the bootstrap iterations described in previous section, 95% confidence limits for *EPL*_*average*_, *EPM*_*average*_ and *EPU*_*average*_ were assessed and for the two compartments combined (lower + middle) when considering Eq (5). In contrast to the length-dependent evaluation, the average values obtained by Eq (6) are specific to the population structures observed during the sea trials and cannot be extrapolated to other scenarios where the size structure of the populations differ.

### Inference of the potential effect on size selectivity in the shrimp fishery

**Fig 3:**
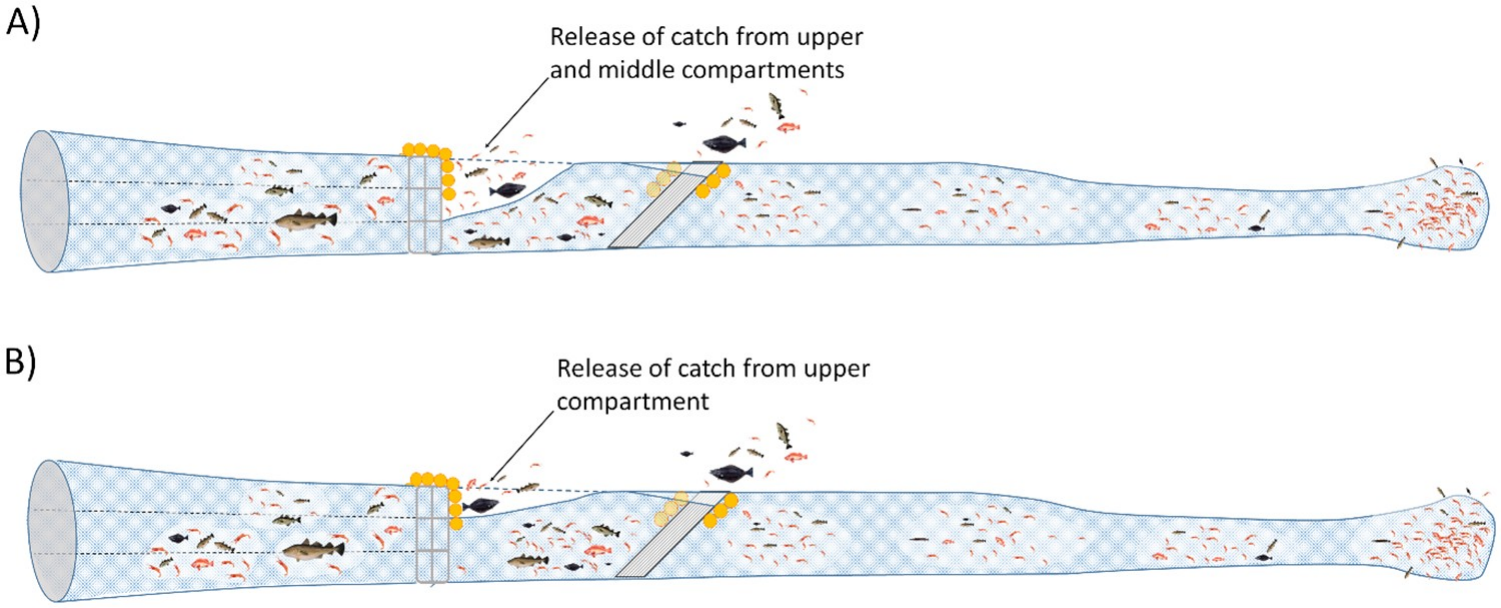
Schematic presentation of two selection systems. The figure is explaining the experimental setup with a vertical separation frame allowing deepwater shrimp and fish to enter the section with the Nordmöre grid and codend from: A) the lower compartment or B) the lower and middle compartments.

To investigate the potential for improving the species and size selectivity in the shrimp trawl with a vertical entry separator frame in front of a Nordmöre grid section, the overall selectivity of the trawl with different configurations at the separator frame was investigated. Specifically, the overall selectivity in the trawl was predicted if the vertical separator was configured so that only shrimp and fish from the lower compartment or from the two lower compartments combined would enter the size selection system. These predictions are valid under the assumption that the insertion of the separator frame does not affect the size selection process of the Nordmöre grid and codend. Fig 3 illustrates these two size selection systems.

Utilizing vertical separation in terms of only directing the shrimp and fish entering the lower compartment *EPL*(*l*) or the lower and middle compartments *EPL*(*l*) + *EPM*(*l*) in front of the Nordmöre grid and codend, the modified overall size selection *rm*_*L*_ (*l*) and *rm*_*L*+*M*_ (*l*) are expected to be:

$$\begin{array}{c} {rm}_{L}(l,vL,\;\vartheta )=EPL(l,vL)\times r(l,\vartheta )\\ {rm}_{L+M}(l,\;vU,\vartheta )=\{1.0-EPU(l,vU)\}\times r(l,\vartheta ) \end{array}$$ (7)

Where we have used Eq 4 and assumed that the vertical separation in the frame and the size selection processes in the aft of the trawl are independent and sequential. This is similar to the prediction approach applied by Melli et al. [39]. *r*(*l*, ***ϑ***) represents the combined size selection of the 19 mm Nordmöre grid and the 35 mm diamond mesh codend (Fig 1) that is compulsory for the Barents Sea shrimp fishery [18]. In Eq 7, previously obtained results for *r*(*l*, ***ϑ***) reported in Larsen et al. [18] and Herrmann et al. [16] were used for the deepwater shrimp and the bycatch species using an identical gear.

Uncertainties in terms of 95% percentile confidence intervals for *rm*_*L*_(*l*, ***vL***, ***ϑ***) and *rm*_*L*+*M*_(*l*, ***vU***, ***ϑ***) were obtained based on the individual bootstrap population for *EPL*(*l*, ***vL***), *EPL*(*l*, ***vU***) and *r*(*l*, ***ϑ***) previously used to estimate uncertainties for these individually. To do this, we estimated the uncertainty for a dual sequential process based on bootstrap populations for uncertainty estimation for individual processes [40]. That is:

$$\begin{array}{cc} \begin{array}{c} {rm}_{L}{\left(l,vL,\;\vartheta \right)}_{i}={EPL(l,vL)}_{i}\times {r(l,\vartheta )}_{i}\\ {rm}_{L+M}{\left(l,\;vU,\vartheta \right)}_{i}=\{1.0-{EPU(l,vU)}_{i}\}\times {r(l,\vartheta )}_{i} \end{array} & i\in \;[1\ldots 1000] \end{array}$$ (8)

Where *i* denotes the bootstrap repetition index. As resampling was random and independent for both groups of results, it is valid to generate the bootstrap population of results for the product based on (6) using two independently generated bootstrap files [40]. The selection curves *rm*_*L*_ (*l*, ***vL***, ***ϑ***) and *rm*_*L*+*M*_ (*l*, ***vU***, ***ϑ***) obtained were then compared to *r*(*l*, ***ϑ***) for each species to evaluate the effect of the vertical separation device in front of the Nordmöre grid. The difference in the length-dependent retention probabilities Δ*r*_*L*_ (*l*, ***vL***, ***ϑ***) and Δ*r*_*L*+*M*_ (*l*, ***vU***, ***ϑ***) were estimated:

$$\begin{array}{c} {\Delta r}_{L}(l,vL,\vartheta ){=rm}_{L}(l,vL,\;\vartheta )-r(l,\vartheta )=\{EPL(l,vL)-1.0\}\times r(l,\vartheta )\\ {\Delta r}_{L+M}(l,vU,\vartheta )={rm}_{L+M}(l,\;vU,\vartheta )-r(l,\vartheta )=-EPU(l,vU)\times r(l,\vartheta ) \end{array}$$ (9)

The 95% confidence intervals for Δ*r*_*L*_ (*l*, ***vL***, ***ϑ***) and Δ*r*_*L+M*_ (*l*, ***vU***, ***ϑ***) were obtained based on the bootstrap population results for *r*(*l*, ***ϑ***), *rm*_*L*_(*l*, ***vL***, ***ϑ***) and *rm*_*L*+*M*_(*l*, ***vU***, ***ϑ***) in a similar way as described above for *rm*_*L*_(*l*, ***vL***, ***ϑ***) and *rm*_*L*+*M*_(*l*, ***vU***, ***ϑ***) [9,19,39].

## Results

### Experimental data

**Fig 4:**
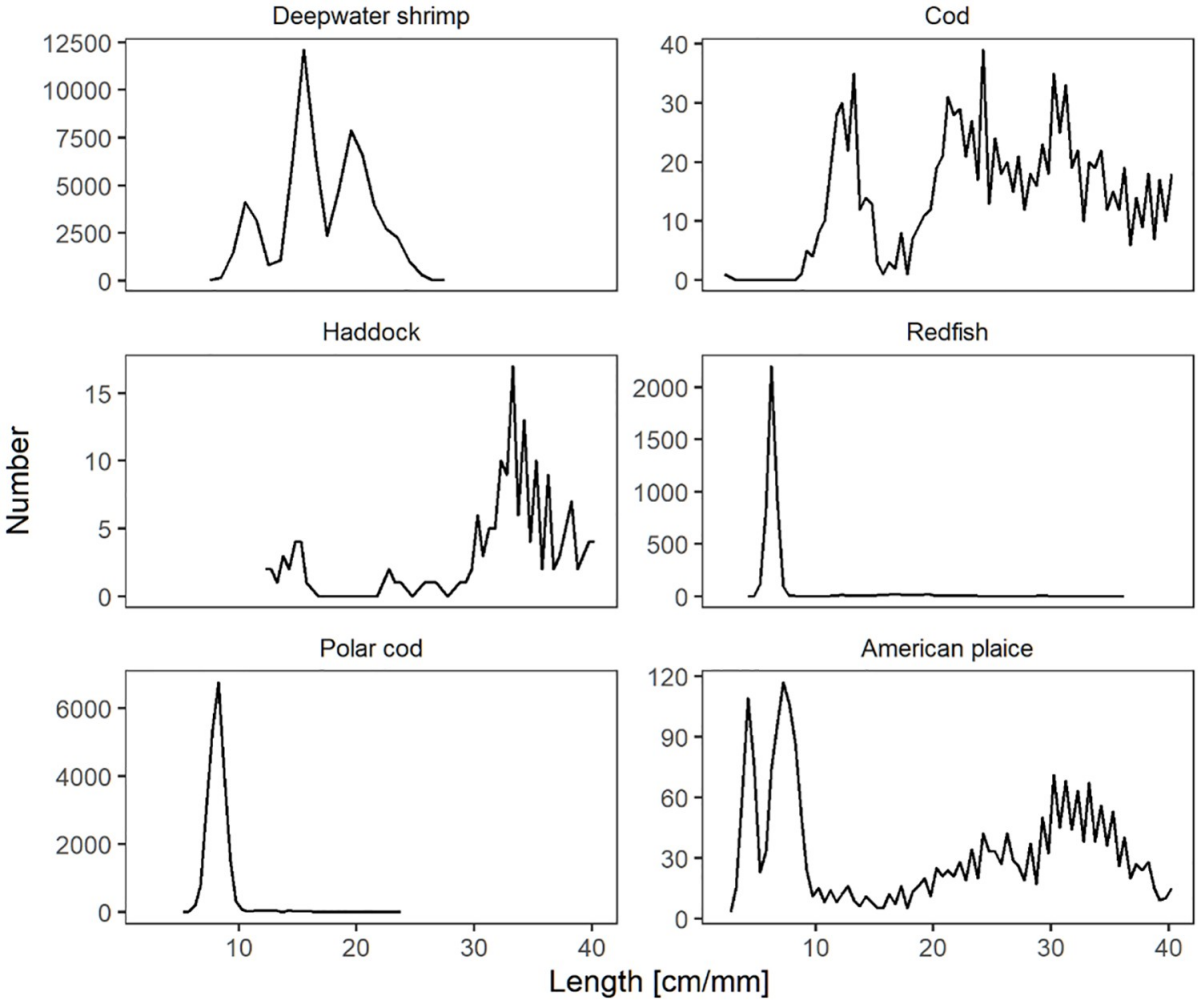
The entry populations of deepwater shrimp and fish. The data was raised according to subsampling ratios and summed over all hauls and compartments for each species included in the analysis.

We conducted 10 hauls in total using the vertical separation device (Fig 2) fishing at depths between 270 and 290 m. The towing speed was within 3.0–3.3 knots. Besides deepwater shrimp the catches contained sufficient number of American plaice, cod, polar cod, redfish and haddock to be included in the investigation (Table 1; Fig 4). The total number of deepwater shrimp, American plaice, cod, polar cod, redfish and haddock being length measured throughout the 10 hauls conducted was 9 028, 2 474, 1 051, 13 811, 4 429 and 155, respectively.

**Table 1:** The number of length-measured individuals.

	**Deepwater shrimp**			**Cod**			**Haddock**		
Haul	Lower	Middle	Upper	Lower	Middle	Upper	Lower	Middle	Upper
1	349 (0.06)	237 (0.06)	264 (0.64)	25	35	18	1	1	1
2	289 (0.13)	297 (0.21)	90 (0.90)	21	51	20	1	4	7
3	369 (0.12)	404 (0.18)	225 (0.93)	23	34	23	0	5	9
4	392 (0.07)	384 (0.12)	203 (0.84)	16	30	19	1	6	6
5	350 (0.08)	291 (0.12)	234 (0.74)	21	47	18	0	6	10
6	498 (0.15)	293 (0.16)	205 (0.80)	43	65	27	3	11	9
7	441 (0.09)	404 (0.17)	185 (0.81)	59	85	41	3	7	12
8	460 (0.10)	333 (0.11)	231 (0.82)	25	44	27	2	4	5
9	340 (0.09)	318 (0.12)	189 (0.71)	32	69	2	2	1	11
10	389 (0.13)	285 (0.24)	79 (0.84)	13	74	44	3	8	16
	**Polar cod**			**Redfish**			**American plaice**		
Haul	Lower	Middle	Upper	Lower	Middle	Upper	Lower	Middle	Upper
1	303 (0.31)	297 (0.11)	528 (0.58)	65	91	83	67	136	50
2	493	1178	444	182	369	249	25	79	27
3	457	1088	364	157	282	221	91	160	45
4	761	342	288	84	103	97	77	152	57
5	510	291 (0.27)	359	82	150	100	57	117	44
6	470	311 (0.26)	541	65	131	143	49	99	25
7	536	482 (0.31)	572	150	279	174	80	129	55
8	413	315 (0.27)	444	83	184	135	111	158	50
9	278 (0.41)	355 (0.21)	338 (0.71)	117	187	155	50	118	34
10	240	592	221	82	132	97	106	179	47

The numbers in parentheses represent the subsampling ratios. In the case where parentheses are not provided, 100% of the catch was length-measured.

**Fig 5:**
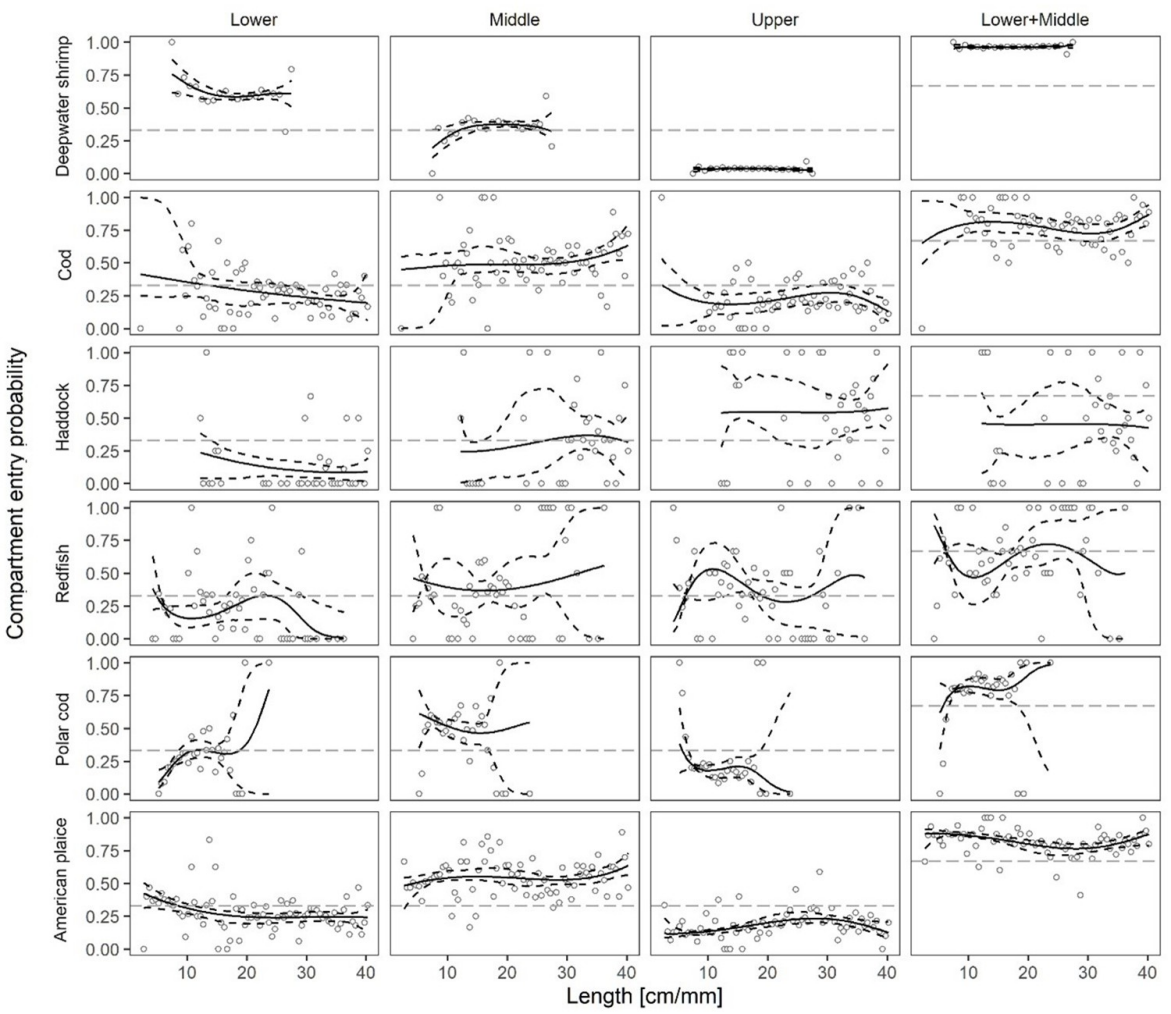
The length-dependent compartment entry probabilities. The figure shows the compartment entry probability for the six observed species for lower, middle, upper and combined lower and middle compartments.

Based on the experimental data (summarized in Table 1), entry probability curves *EPL(l*, ***vL****)*, *EPM(l*, ***vM****)* and *EPU(l*, ***vU****)* were obtained for each compartment (Fig 5). Further, the probability for entering the lower or middle compartment (*EPL(l*, ***vL****)* + *EPM(l*, ***vM****)*) is visualized. Fit statistics in terms of *p*-values and the deviance versus the DOF for the model fit of *EPL(l*, ***vL****)*, *EPM(l*, ***vM****)*, *EPU(l*, ***vU****)* and *EPL(l*, ***vL****)* + *EPM(l*, ***vM****)* are provided in Table 2. For 11 of the 24 cases examined, the *p*-value was below 0.05 and the deviance was much larger than the DOF. However, Fig 5 shows that the models in general follow the main trends in the experimental data, but that there is a considerable amount of dispersion between neighbouring data points. Based on this, we assume that the poor fit statistics noted in the 11 cases is due to over-dispersion in the experimental data rather than an inability for the models to adequately describe the data. Therefore, we are confident in applying the models for *EPL(l*, ***vL****)*, *EPM(l*, ***vM****)* and *EPU(l*, ***vU****)* to quantify the length-dependent probability for deepwater shrimp and the five species of fish to enter in each of the three vertically separated compartments.

**Table 2:** The average compartment entry probabilities *EPL*_*average*_, *EPM*_*average*_ and *EPU*_*average*_ and fit statistics for models *EPL(l*, *vL)*, *EPM(l*, *vM)* and *EPU(l*, *vU)*.

Species	Parameter	Lower (*EPL*)	Middle (*EPM*)	Upper (*EPU*)	Lower + Middle (*EPL*+*EPM*)
Deepwater shrimp	*EPX*_*average*_ (%)	60.3(58.0–63.0)	36.1(33.7–38.0)	3.6(3.1–4.0)	96.4(96.0–96.9)
	*p*-value for *EPX(l)*	0.0019	0.0061	0.2036	0.2036
	Deviance	37.31	33.60	20.38	20.38
	DOF	16	16	16	16
Cod	*EPX*_*average*_ (%)	26.5(21.7–31.2)	50.8(47.0–55.6)	22.7(17.1–27.2)	77.3(72.7–82.6)
	*p*-value for *EPX(l)*	0.0926	0.1349	0.1925	0.1925
	Deviance	74.94	72.17	69.30	69.30
	DOF	60	60	60	60
Haddock	*EPX*_*average*_ (%)	10.3(6.7–13.7)	34.2(27.2–40.7)	55.5(48.2–62.4)	44.5(37.7–51.8)
	*p*-value for *EPX(l)*	0.1438	0.0132	0.0044	0.0044
	Deviance	39.37	51.03	55.49	55.49
	DOF	31	31	31	31
Redfish	*EPX*_*average*_ (%)	24.1(22.7–25.6)	43.1(40.6–44.8)	32.8(31.1–35.2)	67.2(64.7–68.9)
	*p*-value for *EPX(l)*	0.0700	<0.0001	0.0001	0.0001
	Deviance	63.17	116.20	95.80	95.80
	DOF	48	48	48	48
Polar cod	*EPX*_*average*_ (%)	24.5(21.7–30.0)	55.2(49.9–57.8)	20.4(19.2–21.8)	79.6(78.2–80.8)
	*p*-value for *EPX(l)*	0.0944	<0.0001	<0.0001	<0.0001
	Deviance	35.85	105.75	122.32	122.32
	DOF	26	26	26	26
American plaice	*EPX*_*average*_ (%)	28.8(26.1–31.0)	53.6(51.9–55.7)	17.5(16.0–19.2)	82.5(80.7–84.0)
	*p*-value for *EPX(l)*	0.0925	0.0672	0.2453	0.2453
	Deviance	87.24	89.60	78.82	78.82
	DOF	71	71	71	71

The fit statistics are given in terms of the *p*-value and deviance versus the degrees of freedom (DOF). Confidence intervals for the values are given in parentheses.

The curves represent the modelled probabilities *EPL(l*, ***vL****)*, *EPM(l*, ***vM****)*, *EPU(l*, ***vU****)* and *EPL(l*, ***vL****)*+*EPM(l*, ***vM)*** for entry into the lower, middle, upper, and lower + middle compartment, respectively. Stippled curves represent 95% CIs for the modelled probabilities. The circles represent the experimental rates according to Eq (1). The grey horizontal line represents the baseline for no specific vertical entry preference.

Based on Fig 5 and Table 2, the following information regarding the vertical entry pattern of each species is evident.

#### Deepwater shrimp

The majority of deepwater shrimp entered the lower compartment with a length averaged value of 60.3%. This value shows that deepwater shrimp has a strong preference towards the lower panel in the trawl. Further, 36.1% and 3.6% entered the middle and upper compartments, respectively. There is only a weak indication of size dependency in the compartment entry probabilities with a higher proportion of the small (<18 mm) deepwater shrimp entering the lower compartment (Fig 5). Where carapace length >18 mm, the compartment entry probabilities appear to be constant.

#### Cod

The length averaged entry probability values for cod were 26.5%, 50.8% and 22.7% for the lower, middle and upper compartments respectively (Table 2). Smaller cod were more likely to enter the lower compartment (Fig 5) as >33% of cod below 12 cm were estimated to enter the lower compartment.

#### Haddock

Only 10.3% of haddock were likely to be found in the lower compartment, while 34.2% and 55.5% were caught in the middle and upper compartments, respectively (Table 2). There is some indication that the smallest haddock enter the lower compartment, however, low sample sizes prohibit robust analyses of this observation to be made (Fig 5).

#### Redfish

Similar to cod, redfish were more likely to be caught in the middle compartment. The length averaged entry probability values for redfish in the lower, middle and upper compartment was 24.1%, 43.1% and 32.8%, respectively (Table 2). No clear length-dependent entry probability was observed for redfish in the three compartments (Fig 5).

#### Polar cod

As observed for cod and redfish, polar cod were most likely to enter the middle compartment. The length averaged values for the entry probability into the lower, middle and upper compartment were 24.5%, 55.2% and 20.4%, respectively (Table 2). Smallest polar cod had the lowest probability of entering the lower compartment. However, the likelihood of entering the lower compartment increased where size <10 cm (Fig 5).

#### American plaice

American plaice were more likely to enter the middle compartment (Table 2). There was also some indication for small American plaice to enter the lower compartment (Fig 5).

### Predicting the effect on size and species selectivity by utilizing vertical behaviour in trawls

**Fig 6:**
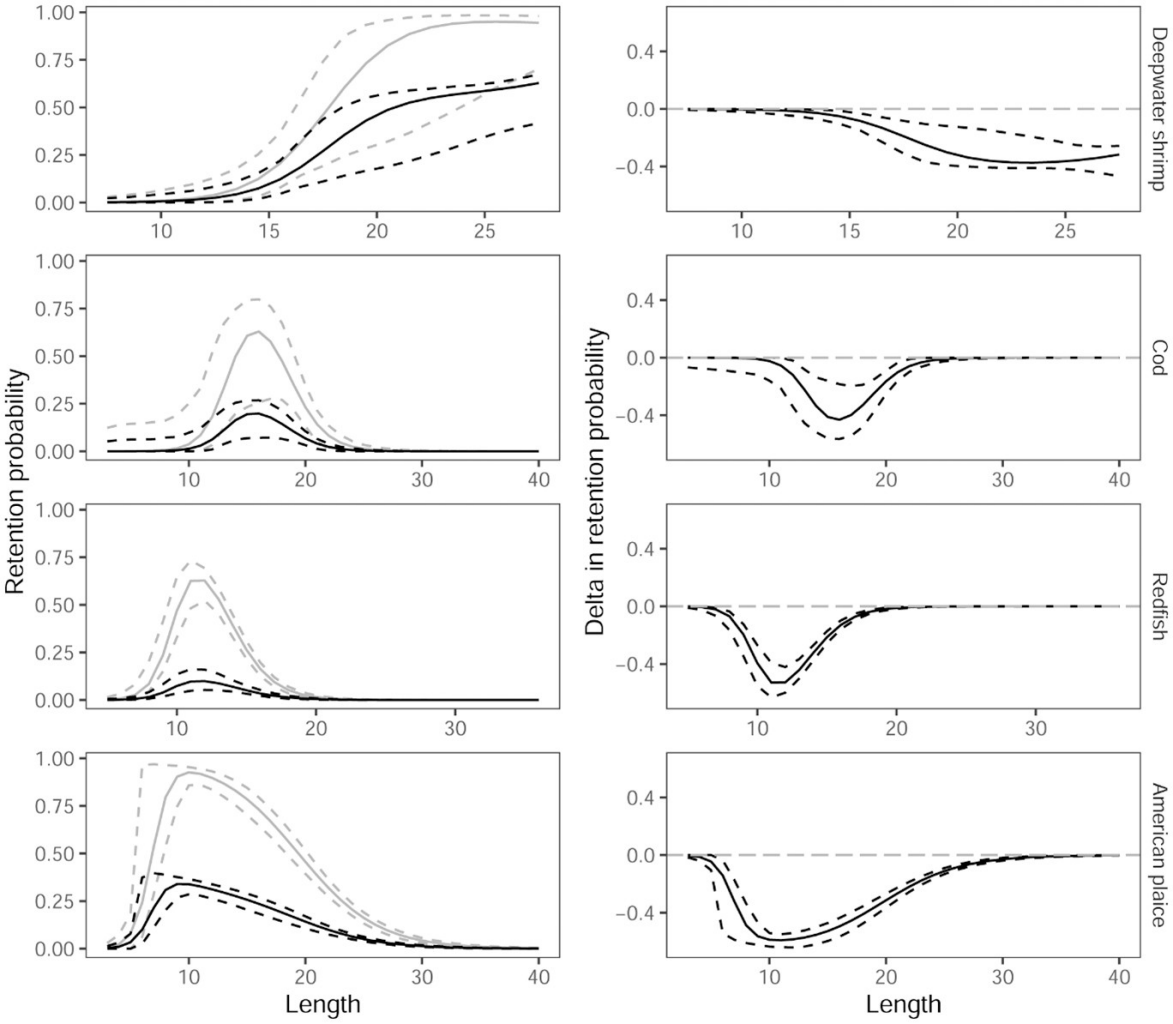
Size selection curves. Left column shows selection curves for deepwater shrimp, cod, redfish and American plaice for the selective system illustrated in Fig 3A (black curves) and for the standard size selection system illustrated in Fig 1 (grey curves). Right column shows the predicted reduction in retention probability if vertical separation (Fig 3A) is utilized. Stippled curves represent 95% CIs.

**Fig 7:**
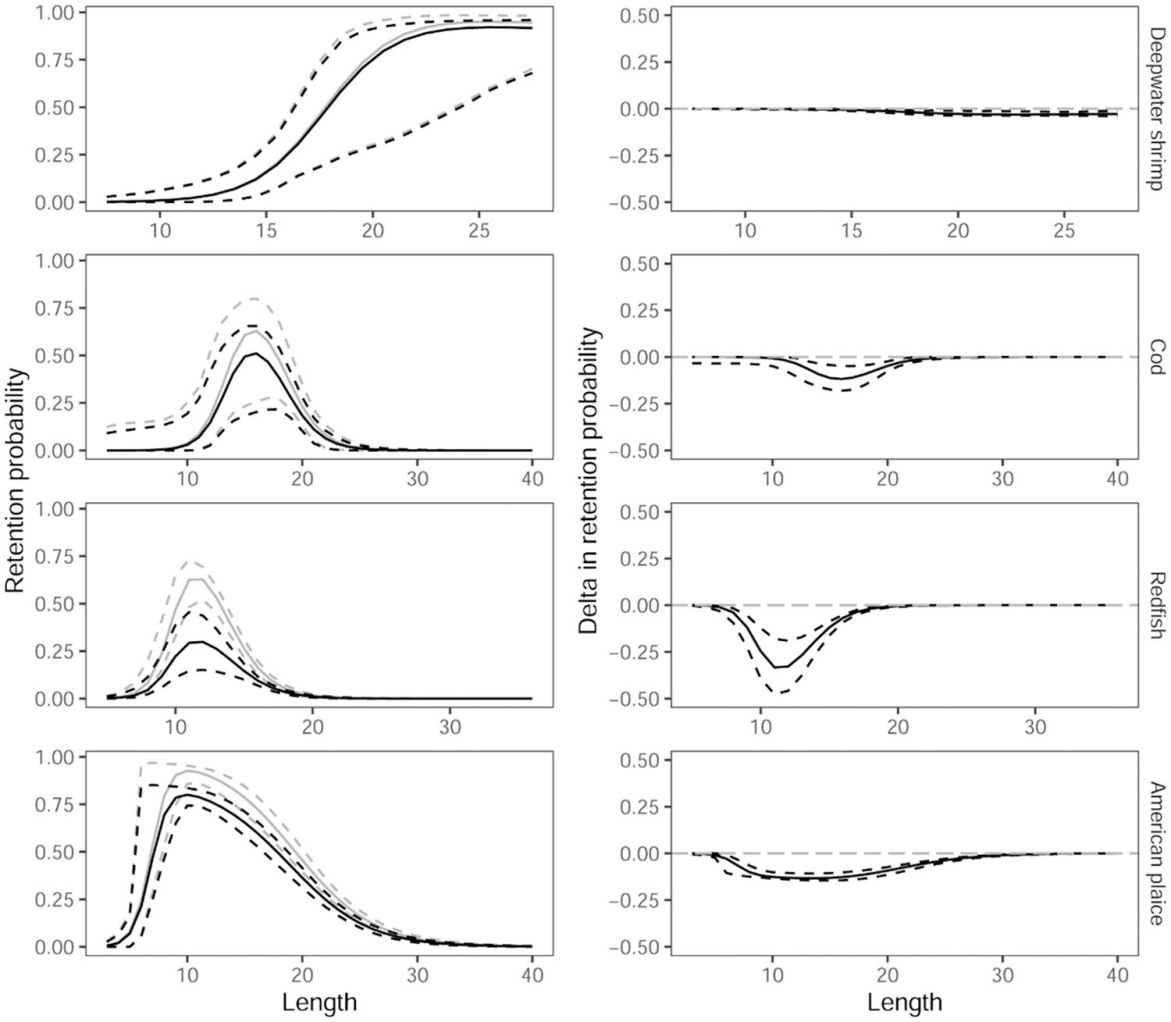
Size selection curves. Left column shows selection curves for deepwater shrimp, cod, redfish and American plaice for the selective system illustrated in Fig 3B (black curves) and for the size selection system illustrated in Fig 1 (grey curves). Right column shows the predicted reduction in retention probability if vertical separation (Fig 3B) is utilized. The stippled curves represent 95% CIs.

By combining the vertical separation results above with previous results on size selection [15,18] in trawls for deepwater shrimp, cod, redfish and American plaice, we were able to predict the size selection curves (Figs 6 and 7) for selective systems shown in Fig 3A and 3B. Predictions for haddock and polar cod were not possible as no data were available for the size selection of these species [16,18].

Significant bycatch reduction in shrimp fisheries is possible (Fig 6) by utilizing the vertical separation (Fig 3A). However, in this case the reduction of the largest and most valuable deepwater shrimp was more than 30% (Fig 6). Since only 3.6% of deepwater shrimp enter the upper compartment (Table 2; Fig 5), it is relevant to examine the size selection of shrimp and fish as illustrated in Fig 3B (Fig 7).

Fig 7 demonstrates that it is possible to utilize vertical separation and maintain nearly the same capture efficiency for the deepwater shrimp while obtaining a significant reduction for several bycatch species with an especially high reduction in the risk of capture for small redfish.

## Discussion

Our results show that deepwater shrimp, American plaice, cod, polar cod, redfish and haddock do not exhibit a uniform vertical entry pattern in the aft of the trawl. Insights of the location of deepwater shrimp and the bycatch species in the trawl before they meet the Nordmöre grid facilitates the use of measures to significantly reduce bycatch in the Barents Sea fishery.

In this study, 60.3% of deepwater shrimp were found to be located in the lower third section of the trawl. This is in accord with previous studies on crustaceans [25,27]. For example, Larsen et al. [41] reported that more than 50% of deepwater shrimp entering the trawl were dispersed within a vertical height of 8 m (when the trawl was within 0–2 m of the seabed), irrespective of size. Unlike in the current study, these authors reported that the majority of deepwater shrimp entering the highest compartments in their net were larger-sized individuals.

Most of the American plaice, redfish, cod and polar cod entered the aft of the trawl in the middle section of the gear, which suggests that these species try to avoid the netting of the upper and lower panels. The results for redfish follow those from previous studies, not exhibiting any vertical preference in the gear [42,43]. However, the results for American plaice and cod contrast with those earlier reported for these two species. Karlsen et al. [27] observed these species to prefer the lower section of the gear rather than the middle, as was observed in this study. Haddock was the only species that showed a different vertical entry preference from the other species examined. More than 55% of the haddock entered the codend through the upper compartment of the frame. This behaviour for haddock in the aft of the trawl has also been reported earlier [27]. All five fish species evaluated in our study were least likely to enter the lowest compartment. The results of the current study indicate that 70% of bycatch could be excluded if the animals entering the two upper-thirds of the gear were directly released. However, this would also result in the loss of more than 40% of deepwater shrimp, which would be unacceptable for fishers and the associated industries. If this direct release of the catch is limited to in the upper third section of the gear, the associated loss of shrimp is predicted to be just 3.6%. Furthermore, in contrast to earlier reported results [41], the entry pattern observed for deepwater shrimp was length-independent, thus there is no indication that this measure would affect the larger, most valuable individuals disproportionately. Assuming that 3.6% is an acceptable loss of deepwater shrimp catch for fishers, the direct release of bycatch from the gear would still be important for species such as haddock (55.5%), cod (22.7%), redfish (32.8%), polar cod (20.4%) and American plaice (17.5%). Polar cod is a species that is considered threatened and of which the bycatch should be avoided [44]. Leaving the upper vertical section of the gear open would also be beneficial for the release of fish above 40 cm, which is often not considered in shrimp selectivity studies [19]. Further, this can improve the performance of the grid by reducing the occurrence of blockages due to for example large flatfish. Our results for the combined frame and grid selectivity are based on predictions that rely on the assumption that inserting a separator frame will not influence the size selectivity of the grid and the codend. Therefore, it is recommended that these predictions are validated experimentally at sea. Further, the trials were carried out during two days in a specific period of the year (December), which could raise concerns regarding how representative these results are for the fishery in general. However, the time of the year and the area in which the experiments were carried out represent rather typical conditions for the commercial fleet. The species caught and the size distributions observed are also expected to be relatively constant over longer periods. Therefore, we assume that our results are representative for comparable shrimp fisheries. Future research should investigate means to direct more of the bycatch to the upper compartment without increasing the loss of shrimp. The use of stimulators such as float lines and chain curtains that cover the path taken by the animals entering the gear have been used to alter the behaviour of fish in the aft of the trawl in the *Nephrops* fishery [30]. The results obtained by Melli et al. [30] showed that such designs of devices can at least partially alter the vertical distribution of fish favourably without changing the vertical distribution of *Nephrops*. If the use of these stimulators would prove to be efficient in the Barents Sea deepwater shrimp fishery, opening the upper compartment would have a substantial impact regarding the reduction of fish bycatch in the fishery.

The proposed design involves both a rigid frame and a sorting grid, which may represent handling issues for the commercial fleet. However, results of this study suggest that the direct release of the catch entering the upper third section of the aft of the trawl should be considered as a valuable bycatch reduction measure in the commercial shrimp fishery.

## Supporting information

S1 Catch Data for individual hauls. The catch data consists of count data for numbers of deepwater shrimp, cod, redfish, polar cod, American plaice, haddock for each of the three compartments. For fish species, “length” corresponds to the total length (in cm) and for shrimp it corresponds to the carapace length (in mm).
(ZIP)
